# Towards the optimal use of video recordings to support the flipped classroom in medical school basic sciences education

**DOI:** 10.1080/10872981.2020.1841406

**Published:** 2020-10-29

**Authors:** Stephen J. Bordes, Donna Walker, Louis Jonathan Modica, Joanne Buckland, Andrew K. Sobering

**Affiliations:** a Department of Biochemistry, St. George’s University, St. George’s, Grenada; b Department of Educational Services, St. George’s University, St. George’s, Grenada

**Keywords:** video, medical education, flipped classroom, basic sciences

## Abstract

The use of recorded video in medical education is increasing. Video material may be assigned before scheduled sessions to create a flipped classroom. Here, the instructor may lead a session that is organized for discussion, interpretation, and reflection of the previewed content. We established conditions that lead to increased student participation and engagement with prerecorded content for a medical genetics section in a first-year medical school basic sciences integrated course. Preliminary analysis of an asynchronous video-based pre-professional program directed the design of video material to support a first semester medical genetics course. We compared student participation in, and opinion of, a flipped-classroom session based on written vs. video presentation of material. Student opinion was surveyed with audience response devices (clickers). Shorter videos that were created specifically for the course were preferred by students compared to recordings of previously delivered lectures. Students preferred videos to assigned reading material and consistent scheduling throughout the teaching semester increased student participation. Presentation of medical school content with previously recorded video material can be a useful teaching tool if properly implemented.

## Introduction

The use of video to supplement content delivery in education programs is rising in popularity [1–3]. Attempts to increase program effectiveness is the main factor contributing to the growing use of video in medical education. Additionally, video supplementation adds to student satisfaction and performance because of its potential to increase active, student-centered learning [4,5]. To exploit this technology, many educational institutions have incorporated a blended learning methodology into their curriculums. One type of combined approach is the flipped classroom which relies on students using asynchronous material and online resources before the lecture [6]. As a result, the flipped classroom is contributing to the modernization of medical education [7].

In the flipped-classroom model, videos and reading assignments released in advance of scheduled class activities enable medical students to prepare more actively for live classroom sessions [8]. Advanced preparation allows for engaging discussions around clinical applications and challenging topics, increased classroom efficiency, and a more thorough understanding of material as learning is both self-paced and focused [6]. The flipped classroom encourages students to prepare for in-class activities more comprehensively by first digesting lecture content outside of the classroom. This allows students time to delve deeper into areas of interest or concern prior to teaching sessions, which increases productivity and understanding of material inside and outside of the lecture hall [9,10].

Goals of the flipped classroom include increasing student pre-lecture preparation and promotion of student engagement with the content. The aim is to engender more robust student participation during class. Research suggests that the flipped classroom creates an environment whereby students attain increased ability to initiate, engage, and lead discussions with peers and facilitators, a task usually left to their professors [11]. Furthermore, audience response devices, such as clickers, can be used by faculty to facilitate classroom peer-to-peer interactions and elicit feedback [9,12]. The flipped classroom also provides opportunities for students to develop and practice active learning strategies while becoming more self-regulated [7]. In the flipped-classroom environment, students must have effective self-regulation and study strategies that support learning independently [8]. These skills include time-management strategies such as fitting the task to the time and focusing. Additionally, the development of active learning strategies such as spaced, layered retrieval practice and integration techniques are encouraged [10].

Here, we examine student perception of a first semester School of Medicine (SOM) genetics lecture session within our curriculum. We compare the effectiveness of the online asynchronous video material to reading assignments. Student perception was ascertained by polling with audience response (clickers) devices. We also compare video length and the inclusion of supporting content such as discussion questions incorporated into case presentations.

## Materials and methods

Online asynchronous video material was created by professors in St. George’s University School of Medicine (SGU SOM) using either lighted screen technology [13] or with Panopto software. Videos created with lighted screen technology were edited with Camtasia software (TechSmith Inc., Okemos, Michigan).

In a pilot study that included 128 students in a small prerequisite SOM course, the content was uploaded to a private channel on YouTube (San Mateo, California) and released in weekly allotments of approximately 10 hours of video to the students. Videos ranged in length from a few minutes to 90 minutes. Data analytics, including number of views and playtime, were gathered from YouTube.

The Panopto (Seattle, Washington) video platform was used to record, host, and collect video lecture data for SOM content. Panopto-created videos were specifically designed for the flipped-classroom lecture that had been carried out in previous terms using only paper cases. Video data collected included the number of hits, length of time watched, and speed at which the video was watched. Survey feedback was collected from first semester medical students regarding preferences on video length using anonymous audience response devices (clickers). All audience response submissions and surveys were gathered using Turning Technologies (Youngstown, Ohio). All surveys were administered during the first of a 2-part flipped-classroom session that occurred during the eighth week of the first semester of medical year 1. All students present in lecture at the time of survey administration were able to participate and personal identifiers such as name, age, ancestry, and sex were not collected. This study was approved by the St. George’s University Institutional Review Board (IRB) and the requirement for consent was waived.

## Results

**Figure 1.**
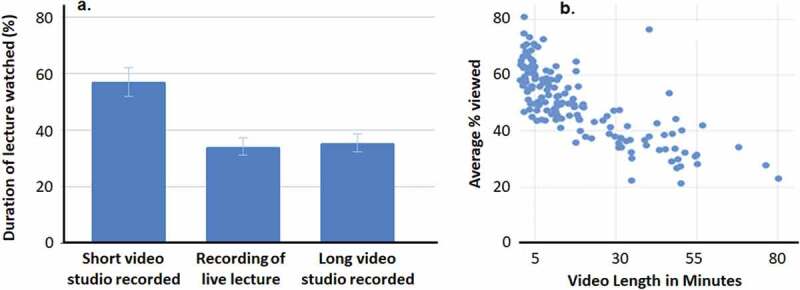
Shorter videos are more likely to be completed in a single take when delivered in our preliminary online science course. Mean average percent of video that was viewed by a student according to type of video (a). Shorter video lengths increase the percentage of the video watched by the student (b)

During our first foray into video lecture presentation creation, all faculty members involved were encouraged to make short, concise segments. However, a significant number of videos were produced in a lengthy lecture format. During the administration of this preliminary content, we were not surprised to find that students were more likely to finish viewing a video without interruption when the length of the video was shorter. Data analytics from YouTube were analyzed after separating all content into one of the three types: (1) short videos that were made solely for the course of 1-minute to 15-minute duration, (2) recordings of previously delivered live lectures that were approximately 50 minutes in length, and (3) lengthy videos that were studio produced solely for online delivery (up to 90-minute duration). Not unexpectedly, we found that students were more likely to view the majority of a video when the length of the content was constrained (Figure 1(a)). From 61,778 views of students who were enrolled in the course, we plotted how much of a video was viewed as a function of the length of the video in minutes. When analyzed this way, we saw that up to 80% of a video was likely to be viewed when in a short format. Longer videos were much less likely to be viewed in their entirety (Figure 1(b)).

**Figure 2.**
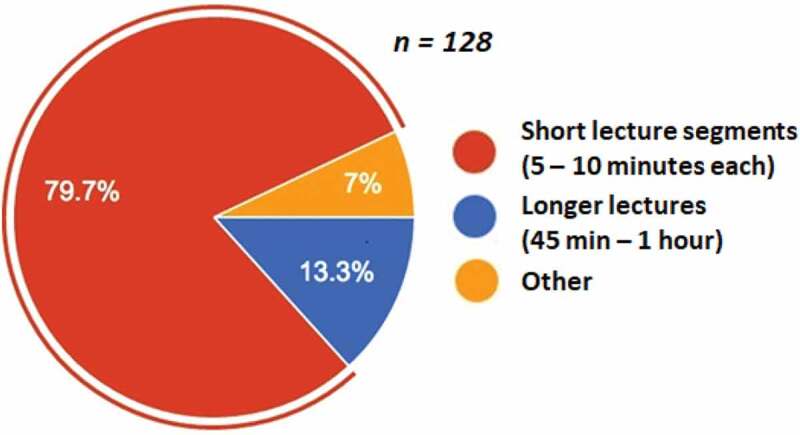
Survey of student opinion about video length in our preliminary online science course. Short videos are preferred (*n = 128 student responses*)

Since we noticed a difference in the way video content was consumed, we directly asked the students in this preliminary cohort about their personal preferences regarding the video format. Of the 128 responses, most reported a preference for shorter videos. Only 13.3% of respondents indicated a preference for longer lectures. Some other options would have been preferred by 7% of the respondents (Figure 2). Open comments from this survey revealed that students found the asynchronous video format to be a good experience, and the online media provided was perceived as beneficial as it allowed coursework to be attended to according to individual schedules.

Encouraged by the experience of this preliminary use of video, we decided to support teaching in the SOM with a similar model. We developed videos for a first semester medical genetics lecture that was previously structured using a flipped-classroom model with pre-readings and assigned discussion questions only. This flipped-classroom session was always scheduled in week 8 of the first semester of medical year 1. In all iterations of the flipped class, students were told that the session would not be graded. Instead, students were told that preview of the assigned material would ensure that the live portion of the activity would be more useful to them. Classroom interaction was facilitated with anonymous audience response devices (clickers) and multiple-choice questions (MCQs) that were based on vignettes extended from the pre-assigned material. Because of this design, the instructor was able to discuss and interpret the pre-assigned material during the scheduled class session. Our flipped-classroom session is not a simple review session as we also teach new concepts and connections following the clicker questions that were based on the previewed material. In this way, we see the clicker questions as a bridge between the content included in the preassigned videos, and the eventual attainment of the course objectives.

During the session, we also anonymously queried students about their opinions and perceptions of the flipped classroom. We asked the students if they (A) enjoyed the session and would like to see more lectures delivered this way or (B) have the traditional lecture instead. By this survey, we found that students almost unanimously reported that they appreciated the session when it was set up with reading assignments only (Spring 2017 and Fall 2017). However, students also admitted that they were unlikely to prepare for the session. Beginning in the Spring 2017, we anonymously asked how much time students spent preparing for the session with the following options: (A) Zero minutes, (B) 5 minutes, (C) 10 minutes, (D) 30 minutes, and (E) 60 minutes or more. Responses from the class showed that 24.6% of the students spent 30 minutes or more of preparation time with the assigned reading or discussion questions in the Spring 2017 semester. Importantly, almost half of the students reported that they did not prepare for the session at all. We saw a similar distribution of responses in the Fall 2017 semester.

Further, the majority of the students claimed that they would be more likely to prepare if video segments were provided as pre-lecture material in addition to only being given a reading assignment with discussion questions. Our students told us that if we were to include video content, they would increase their preparation, participation, and most importantly, their buy-in for the flipped-classroom session. In response, we created videos for the next semester (Spring 2018) to supplement the assigned readings. Since our objective was to increase student engagement, we again asked our students how the addition of video content impacted their preparation for the flipped-classroom session. Surprisingly, we found that the proportion of students who prepared for the flipped-classroom session did not increase – the introduction of video did not induce the students to prepare for the session.

**Figure 3.**
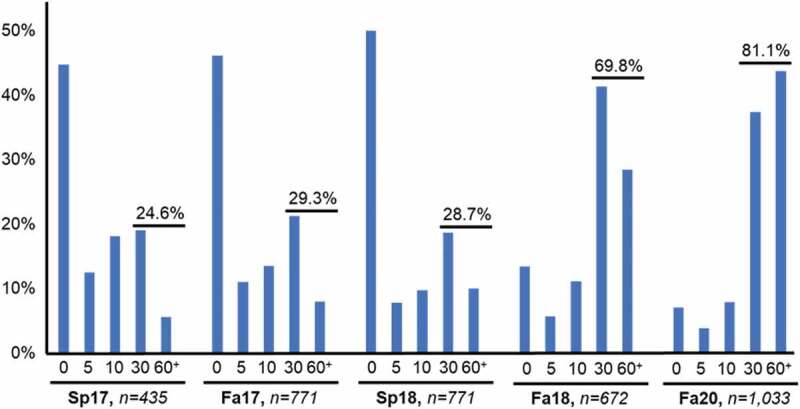
Percentage of students who report spending 0, 5, 10, 30, or 60 plus minutes preparing for the flipped classroom (*n* = *number of students who responded*). In Sp17 (spring) and Fa17 (fall), the preparatory material consisted solely of assigned readings and discussion questions. In Sp18, videos were included. In Fa18, video assignments were consistently scheduled throughout the semester. In the Fa20 semester, our response to the global COVD-19 pandemic forced us to deliver all course material online

The subsequent SOM semester (Fall 2018) incurred a major curricular change in which approximately 33% of all passively delivered lecture content was removed and replaced by directed learning activities (DLA). The assigned content consisted predominantly of video segments accompanied by some required readings and non-video tutorials. Importantly, the programming of the content was consistently distributed throughout the term, beginning on the first day of the semester. During this offering, the genetics flipped classroom lectures in week 8 remained the only example of this type of teaching session in the curriculum. However, because of the regularly assigned videos, we found a large increase in the number of students who prepared for our flipped-classroom activity. Because of the global response to the COVID-19 pandemic, our Fall 2020 semester was moved to an entirely online delivery system and we saw more than a 10% increase in participation (Figure 3).

**Figure 4.**
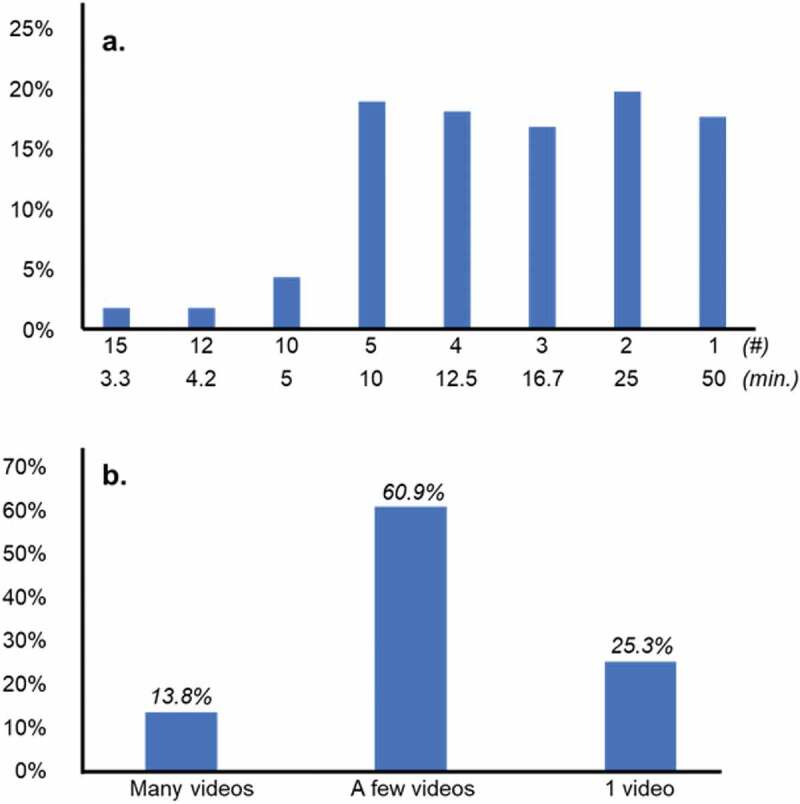
Video length preference among students in a year one medical school course. (a) In the Spring 2020 semester, students were polled and asked what length of video would best suit their learning style: 15 videos at 3.33 minutes each, 12 videos at 4.17 minutes each, 10 videos at 5 minutes each, 5 videos at 10 minutes each, 4 videos at 12.5 minutes each, 3 videos at 16.7 minutes each, 2 videos at 25 minutes each, or a single video that is 50 minutes long *(# = number of videos; min. = approximate minutes per video; n = 473 student responses*). (b) In the Fall 2020 semester, students were polled and asked if they would prefer many videos, each about 1–5 minutes long, a few videos, each about 10–20 minutes long, or a single video that was 50 minutes in length (*n = 1,031 student responses*)

In the Spring 2020 semester, we queried students for their opinion regarding the optimal time-length of video that they would find most useful in their studies. We introduced the query by asking students to imagine a typical lecture that would be 50 minutes in length and to consider the optimal way that the content should be packaged into the video. From this, we found that most students would be happy with a range of video lengths, but only a few preferred very short or very long videos (Figure 4(a)). Because of the heterogeneity of the responses from the Spring 2020 term, we redesigned the survey question for the following (Fall 2020) term. We simply asked the students if they would prefer many videos, each about 1 to 5 minutes in length; a few videos each about 10–20 minutes in length; or a single video, of approximately 50 minutes in length. We were not surprised to find that most students (60.9%) reported a preference for the content of medium length (Figure 4(b)).

**Figure 5.**
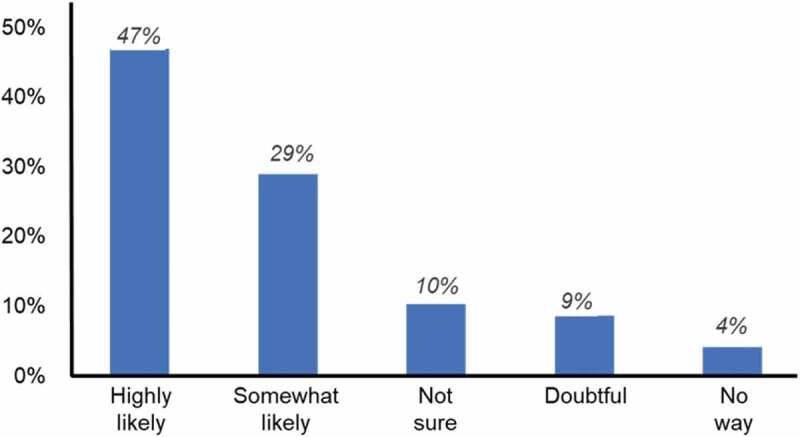
Student survey regarding inclination to revisit assigned material following a flipped-classroom session. The majority of students intend to review assigned material (*n = 666 student responses*)

We also saw that the use of audience response devices in the flipped classroom had an impact on the self-regulated learning behavior of our students. We surveyed the Fall 2018 cohort of students regarding their perceptions of the session. We asked the students to tell us, based on their experience with our flipped classroom, if they would return and review the assigned material. Survey options were as follows: Highly likely, Somewhat likely, Not sure, Doubtful, and No way. We found that more than 75% of the students reported that they were either highly likely or somewhat likely to review the material following the session (Figure 5). These findings indicate that using MCQs with audience response devices in a flipped classroom promotes the development of self-regulation in medical students [8].

## Discussion

Asynchronous online content used to support lectures, small group discussions, and other class activities is well established, and its positive impact on teaching and learning is upheld by our findings. Interest in the flipped-classroom approach has led many medical schools to adopt a supplementary delivery platform for students using a variety of different media [7]. Asynchronous content should be comprehensive, easily accessible, and made available to students in a timely manner. As a result, certain limitations may exist if schools lack resources necessary for the preparation of instructional videos such as support for faculty and software that is user-friendly for recording, editing, and publishing [14].

### Video length

With the majority of student respondents indicating their preference for shorter bursts of asynchronous online content, we conclude that both content organization and viewer satisfaction improve when presenting a topic in shorter video lengths. Our conclusions agree with previous studies in which a flipped classroom was tested in a second semester medical biochemistry course [15] and from analysis of video content from massive open online courses [16]. Students are drawn to material that improves their learning efficiency and motivates them both intrinsically and extrinsically [17]. Material that quickly conveys key concepts in a short amount of time is preferred over material that must be sorted through and digested slowly. Furthermore, the resulting increase in interactive class time positively correlates with student satisfaction [1]. We interpret these findings as an indication that shorter videos assist students in their organization. The focused and concise video format also helps students to better integrate the material within the context of the overall curriculum.

In our study, we found that videos of 10- to 20-minute duration were highly favored by students. We feel that this is the optimal length range for an instructional video as it allows for a better conceptual framing of course objectives. Additionally, we feel that content organization and topic development may be maximized in that amount of time. Anecdotal observations suggest that very short videos (i.e., videos that are 5 minutes or less) were less favorably received by students as they usually were bundled together as multiple files. Therefore, students and faculty course directors had the increased burden of organizing and keeping track of files and materials, three- to fourfold in number. The rate of upload and dissemination error also increased as a result.

Additionally, very short videos may not be long enough to convey meaningful information. Students also tend to overestimate the amount they can accomplish in the time they have allotted for learning activities [18]. As such, we believe that the onus of responsibility falls on course directors to organize material in such a way that maximizes the time and efficiency of the student. Higher quality content and organization will, in turn, encourage a larger viewership, increase participation among the student body, and lead to a greater understanding of content objectives [19]. An important aspect when implementing this type of teaching paradigm is the increased level of organization and predevelopment of outcome objectives prior to running the course [20]. Others have noted the essential nature of creating, and maintaining, a high level of structure and organization within interactive learning programs [21]. We extend these conclusions by asserting that this responsibility is even more important when integrating electronic media to support teaching in the medical school curriculum.

### Video as part of the curriculum

We note that other studies have shown that the flipped-classroom model is efficacious and can support traditional methods in medical education [15,22]. One study showed that videos hosted on YouTube can support problem-based learning (PBL) sessions for anatomy [23]. Other work has shown student participation with online video content can be problematic, and that students tend to preferentially access videos when they sense that they are having difficulty with the material [24]. From our studies, we have found that students are more likely to prepare and view the videos when there is a consistent offering of video content throughout the semester.

The use of multimedia of many different types can be a powerful substitution or supplementation to the live lecture [25,26]. In general, the use of technology-leveraged content is steadily increasing among faculty who teach in higher education [27,28]. We feel that with proper integration into the education curriculum, video, either hosted on the course support platform, YouTube, or elsewhere, and other forms of technology including social media have the potential to transform our educational content and our interactions with our students.

### Study limitations

As with all research on educational practices, the interpretation of our study has multiple limitations. Analytics collected from videos posted on YouTube rely on the vendor's data which can potentially provide incomplete/skewed data. If students access these videos offline (download them) or with a VPN, this data can be missing or incomplete. Other vendors such as Panopto provide a bit more of a controlled environment since students must use university credentials to access the content and the platform records more specific data. Even with the use of contracted vendors such as Panopto, tech-savvy students may still discover methods to download the videos. Additionally, we are unable to ascertain if students viewing video recordings accelerate the viewing process and to what extent they do so.

Studies that follow an entire curriculum cannot control for the quality and charisma of individual lecturers. Some presenters have a stronger ability to keep students engaged. Future studies could be designed to attempt to control for lecturer quality and can compare longer or shorter lecture segments created by the same instructor or attempt to quantify the ability of that instructor to keep students engaged. Other aspects of the lecturer that could affect student’s willingness to watch the recording were not controlled for include: audio quality, number of slides given to students, and student’s difficulty/familiarity of the topic (newer information compared to continuation of a lecture series).

We looked at the sum total number of engagements with all videos that were used in our course, which reflected a large size in our study. When analyzed in aggregate, we see the trend that shorter videos maintain more consistent attention from our students based on video completion in a single take. A future study could collate the total number of videos analyzed per individual lecturer over several course offerings. This approach may provide data to control for the aforementioned variance in lecturer quality.

The purpose of this study is to determine student engagement and satisfaction with video delivery within the context of the flipped-classroom format. However, we are unable to describe the actual effectiveness of our approach in terms of student performance for several reasons. One is that it is difficult to compare exam performance from one semester to another as individual differences in the exam questions used create too many confounding variables. A second reason is that during the semesters where we were collecting our data, our curriculum was undergoing substantial changes. This curricular evolution is actively creating additional confounding variables.

An example of a successful study where students within a course were randomly split into two cohorts showed that students participating in active learning score higher: enrolled students were either placed into a course that was based on an innovative active approach or the traditional passive lecture approach. This study showed a robust increase in student performance with active learning methods [29]. Other studies noted the difficulties and confounders associated with attempting to split a course offering into control and experimental cohorts [30]. Both of the studies described above were done with Bachelor of Science level science classes. The increased stakes of a preprofessional medical school curriculum increase the difficulties in study design, so attempting to answer the question of quality improvement becomes much more difficult.

## Conclusion

The integration of asynchronous lecture content and the flipped-classroom model are well liked by students who are enrolled in the medical curriculum at SGU. Initially, the creation of video was viewed as a daunting task by the faculty. However, with the introduction of user-friendly technology, the creation of such material can be relatively straightforward for faculty members when given access to the appropriate tools and training. The feedback we received from students is largely positive and agrees with previous reports describing online lectures [6]. Our findings complement previous research on the flipped classroom which conclude that students who have positive, meaningful interactions with the content develop their own learning processes, resulting in improved self-regulation and long-term retention [31]. Once students develop effective strategies to approach learning activity objectives, they are better able to manage study time efficaciously, thoroughly engage with video content and its online delivery platform, and self-assess knowledge gaps using audience response feedback and other forms of assessment such as formative quizzes [8].

In this study, we reached three major conclusions. First, we showed that the utility of the flipped classroom is maximized when video content is consistently delivered throughout the semester as we see participation nearly triple when the overall curriculum is designed in this manner. Second, we observed that the use of clicker questions in the flipped classroom increases student awareness of course content. This awareness provided impetus for students to revisit the material and thus helps to promote self-regulated behavior. Finally, our findings showed that students appreciate videos that are neither too long nor too short, and thus recommend use of videos from 10 to 20 minutes in length.

Future studies on video use in medical education might focus on how students use the information contained in educational videos. Foci of study might include the timing as to when students actually view the material or changes in viewership just prior to summative exams. Additional studies might look at how many students view the material at increased speed. Other areas of qualitative inquiries may be attempting to understand how students interact with the video material. For instance, are students motivated to take notes, create concept maps, and generate their own study material (lists/tables) from video presentations or are videos just another substitute for the passive lecture?

During the ongoing COVID-19 pandemic, the use of video and the video presentation of lecture material has increased dramatically. As the response to this pandemic evolves, it is likely that medical school basic sciences curricula will remain altered. Challenges to medical education teaching methodology in this time are therefore numerous. The way that we implement the use of video recordings in our respective curricula needs careful thought to be optimally successful.

## Abbreviations

DLA (Directed Learning Activity).
